# Sudden Cardiac Death in Patients with Heart Disease and Preserved Systolic Function: Current Options for Risk Stratification

**DOI:** 10.3390/jcm10091823

**Published:** 2021-04-22

**Authors:** Luigi Pannone, Giulio Falasconi, Lorenzo Cianfanelli, Luca Baldetti, Francesco Moroni, Roberto Spoladore, Pasquale Vergara

**Affiliations:** 1 Cardiology Department, IRCCS San Raffaele Scientific Institute, 20132 Milan, Italy; pannone.luigi@hsr.it (L.P.); giuliofalasconi@gmail.com (G.F.); cianfanelli.lorenzo@hsr.it (L.C.); baldetti.luca@hsr.it (L.B.); moroni.francesco@hsr.it (F.M.); ambulatorio.rscardiologia@gmail.com (R.S.); 2 Arrhythmia Unit and Electrophysiology Laboratory, IRCCS San Raffaele Scientific Institute, 20132 Milan, Italy

**Keywords:** sudden cardiac death, preserved ejection fraction, ICD, sudden cardiac arrest

## Abstract

Sudden cardiac death (SCD) is the leading cause of cardiovascular mortality in patients with coronary artery disease without severe systolic dysfunction and in heart failure with preserved ejection fraction. From a global health perspective, while risk may be lower, the absolute number of SCDs in patients with left ventricle ejection fraction >35% is higher than in those with severely reduced left ventricle ejection fraction (defined as ≤35%). Despite these observations and the high amount of available data, to date there are no clear recommendations to reduce the sudden cardiac death burden in the population with mid-range or preserved left ventricle ejection fraction. Ongoing improvements in risk stratification based on electrophysiological and imaging techniques point towards a more precise identification of patients who would benefit from ICD implantation, which is still an unmet need in this subset of patients. The aim of this review is to provide a state-of-the-art approach in sudden cardiac death risk stratification of patients with mid-range and preserved left ventricular ejection fraction and one of the following etiologies: ischemic cardiomyopathy, heart failure, atrial fibrillation or myocarditis.

## 1. Introduction

Sudden cardiac death (SCD) is an event of presumed cardiac origin occurring suddenly and unexpectedly in an otherwise stable patient [1]. From a healthcare perspective, with a rate as high as 183,000 deaths per year, SCD represents a major social issue [2]. Despite several improvements in the treatment of cardiovascular diseases, SCD still accounts for 2.04 million or 40–50% of the potential years of life lost [2,3]. Among men, death rate from SCD (76 per 100,000) exceeds all other individual causes of death including lung cancer, accident, chronic lower respiratory disease, cerebrovascular disease, diabetes mellitus, prostate cancer and colorectal cancer [3].

The implantable cardioverter defibrillator (ICD) represented a turning point in the prevention of SCD, with several landmark trials demonstrating its efficacy in selected populations [4,5]. Building on these studies, ICD is currently recommended to reduce the risk of death in patients with severely reduced (≤35%) left ventricle ejection fraction (LVEF) (primary prevention) and in cardiac arrest survivors (secondary prevention) [6,7]. Notably, while patients with LVEF ≤ 35% are at the highest absolute risk of death, more than 70% of SCD in patients with coronary artery disease (CAD) occur in patients with LVEF >35%, leaving most subjects at risk largely uncaptured by an LVEF-centered risk stratification [8]. Furthermore, SCD is the most common cause of cardiovascular death in patients with CAD without severe systolic dysfunction and in patients with heart failure with preserved ejection fraction (HFpEF [9]). While absolute risk may be low in the LVEF > 35% population, the large number of patients at risk and the devastating consequences of SCD pose an intriguing clinical challenge.

**Figure 1:**
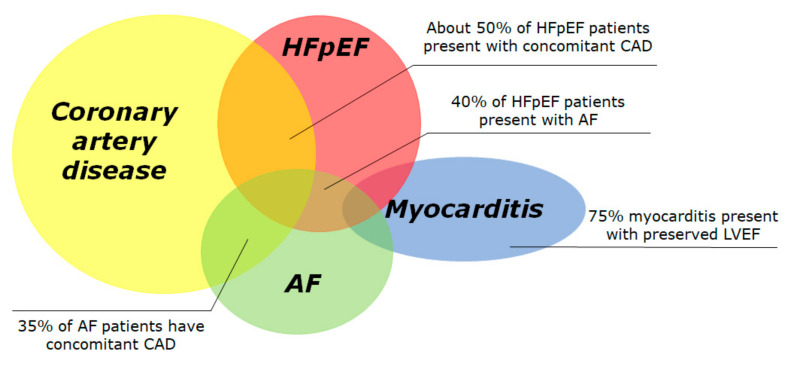
Prevalence of conditions associated with preserved left ventricular ejection fraction and increased risk of sudden cardiac death. The Venn diagram displays the four most frequent conditions associated to sudden cardiac death, i.e., coronary artery disease, heart failure with preserved ejection fraction, atrial fibrillation and acute myocarditis. Many conditions overlap with others. The overall incidence of SCD in each subgroup is largely unknown due to difficult data collection. AF: atrial fibrillation; HFpEF: heart failure with preserved ejection fraction.

In the following sections, we will review the current approach to risk stratification of SCD in the population with LVEF > 35%, in the four most common subsets: CAD, HFpEF, atrial fibrillation (AF) and myocarditis (Figure 1). In the real world, significant overlap exists between these conditions, since 50% of HFpEF patients present with concomitant CAD [10,11], 40% of HFpEF patients present with AF [12] and 35% of AF patients have concomitant CAD [13]. We reported about currently published studies and clinical trials, selected after a systematic research on PubMed including the keywords “sudden cardiac death” and “preserved” or “mid-range”.

Other conditions at high risk of SCD with preserved ejection fraction, such as idiopathic dilated cardiomyopathy, hypertrophic cardiomyopathy (HCM), arrhythmogenic right ventricular cardiomyopathy (ARVC), channelopathies and valvular diseases, are beyond the scope of this paper.

## 2. Epidemiology

Several registries and Clinical Trials evaluated, with different approaches, the relationship between LVEF and incidence of SCD (Table 1). The observational Maastricht Circulatory Arrest Registry [14] retrospectively evaluated 2019 patients from 1997 until 2000. In this registry, SCD accounted for 19% of total mortality in the age group between 20 and 75 years, and CAD was the most frequent underlying cause of death. Echocardiographic data were available for 200 patients. The SCD rate was higher among patients with lower LVEF, from 7.5% in patients with LVEF < 30% to 1.4% in patients with LVEF >50%. However, the absolute number of SCD victims was higher in the group of patients with LVEF >50%.

Similar results came from the retrospective Oregon Sudden Unexpected Death Study, reporting on all SCD cases in Multnomah County (Oregon) between 2002 and 2004 [15]. The authors labelled LVEF as severely reduced, mildly reduced or normal in the presence of LVEF <35%, between 35 and 54% and >55%, respectively. The LVEF was severely reduced in 36 (30%) patients experiencing SCD, mildly reduced in 27 (22%) and normal in 58 (48%). Even accounting for history of resuscitated cardiac arrest, long QT or Brugada syndromes, HCM and ARVC, only 35% of effective SCD cases would have been labeled as patients at high risk of SCD. In other words, 65% of SCD patients in this cohort would have been considered at low risk of cardiac death and not considered for primary SCD prevention with ICD.

## 3. Risk Stratification of SCD in Patients after Myocardial Infarction without Severe Left Ventricular Dysfunction

The VALIANT trial [1] evaluating valsartan, captopril or both in 14,703 patients with acute myocardial infarction (AMI) is the largest report investigating SCD in post AMI patients. Patients were enrolled between December 1998 and June 2001, and the median duration of follow-up was 24.7 months. Of 14,609 patients, 1067 (7%) experienced SCD. The SCD rate was 10-fold higher in the first 30 days after MI (1.4%), decreasing exponentially over the first 6 months and reaching a steady rate of 0.14%/month at 2 years. SCD was the cause of death in 10% of patients with LVEF ≤30%, as compared to 6% in patients with LVEF of 31–40%, and 5% in those with LVEF ≥40%. Each decrease of 5% points in LVEF was associated with a 21% increase in the risk of SCD.

These results were also confirmed in the PRE-DETERMINE study [8], a prospective observational cohort study including 5761 participants with CAD and LVEF >35%, or 30–35% plus NYHA class I. During a median of approximately 4 years, the cumulative incidence of SCD was 2.1%. The 4-year cumulative incidence of SCD was 1% in patients with LVEF >60%, 1.6% in patients with LVEF 50–59%, 3.2% in patients with LVEF 40–49%, 4.9% in patients with LVEF 30–39%. Each 10% decline in LVEF was associated with a 71% increase in the incidence of SCD. A clear association between lower LVEF and SCD risk emerged clearly from these observational and randomized studies: the striking higher relative risk of patients with severely reduced LVEF may have obscured and contributed to the underestimation of the SCD risk in the LVEF > 35% population.

Risk stratification for SCD in patients with previous MI and preserved LVEF (pLVEF) has been explored in the PEACE study [16], where clinical variables associated with a higher proportional risk of SCD were angina pectoris, LVEF > 40% but <50% (as opposed to >50%), diuretics use, digitalis use, prior coronary revascularization and being female or Caucasian. These variables were confirmed to a certain extent in the PRE-DETERMINE study [8], with age <60 years, LVEF 30–49% and diabetes mellitus being associated to SCD. Several other invasive and non-invasive methods have been suggested to enhance SCD risk stratification in post-MI patients. Simple clinical variables that may warrant ICD implantation can be obtained at time of MI hospitalization. In the DAPA trial [22], ICD implantation was associated with a net mortality benefit in patients after primary percutaneous coronary intervention for ST-segment–elevation myocardial infarction, if at least one among the following risk factors was present: LVEF <30% within 4 days, final TIMI flow < 3 after primary PCI, primary VF, Killip class ≥ 2. Notably, at 18 months follow-up half of patients enrolled had LVEF >30%.

### 3.1. Basal ECG

Valuable information can be derived from careful analysis of the basal ECG. Based on the Oregon Sudden Unexpected Death Study, an ECG-based risk score for SCD stratification in patients with pLVEF was developed [23]. The score encompasses heart rate, criteria of LV hypertrophy, precordial QRS transition lead, QRS-T axis, QTc and time between Tpeak and Tend. This tool was externally validated in the Atherosclerosis Risk in Communities (ARIC) study [23]: in this cohort, subjects with ≥4 ECG abnormalities had an odds ratio (OR) of 21.2 (CI 9.4–47.7; *p* < 0.001) for SCD. In the LVEF >35% subgroup, the OR was 26.1 (CI 9.9–68.5; *p* < 0.001) for the high risk group (≥4 ECG abnormalities). The addition of the ECG risk score to a multivariate model including LVEF, age, sex, hypertension and diabetes increased the C-statistic for SCD from 0.625 to 0.753 (*p* < 0.001), with net reclassification improvement of 0.319 (*p* < 0.001). In the ARIC cohort validation, risk of SCD associated with ≥ 4 ECG abnormalities remained significant after multivariable adjustment (HR 4.84; *p* < 0.001; C-statistic improvement from 0.759 to 0.774; *p* = 0.019).

### 3.2. Autonomic Dysfunction

Microvolt T-wave alternans (TWA) is a beat-to-beat fluctuation in the amplitude or shape of T waves. Microvolt TWA is not visually detectable on continuous ECG but can be measured during bicycle or treadmill exercise with spectral analysis [24]. First experiences in the canine model [25] demonstrated that increasing TWA was associated with lower ventricular fibrillation threshold.

In a prospective observational study by Ikeda et al. [18], 1041 post-MI patients with an LVEF ≥ 40% (average 55 ± 10%) TWA were analyzed a mean of 48 days after acute MI. Presence of TWA, non-sustained ventricular tachycardia (NSVT), ventricular late-potentials (LP) and lack of coronary revascularization were associated with SCD or life-threatening arrhythmias. Interestingly LVEF was not a predictor of SCD in this study: inclusion of patients with LVEF ≥40% may explain these findings, underpinning the lower weight of LVEF in the cohort of patients without severely reduced LVEF as a SCD prognosticator.

Other measures of autonomic dysfunction as heart rate turbulence (HRT) and deceleration capacity (DC) can be obtained from 24-h Holter ECG recordings. HRT quantifies the physiological short-term oscillation of cardiac cycle lengths that follows spontaneous premature ventricular complexes (PVC). HRT consists typically of a brief heart rate acceleration followed by a gradual heart rate deceleration. DC is an integral measure of all deceleration-related oscillations observed over 24 h. Together, abnormal HRT and DC are markers of severe autonomic failure (SAF).

In the REFINE study [26], post-MI patients who developed the primary outcome of cardiac death or cardiac arrest had significantly lower LVEF (38 vs. 40% at 7-day and 40 vs. 49% at 8-week; *p* < 0.01) and smaller increases in LVEF than the remaining patients (+2 vs. +9% at 8-week). Furthermore, history of diabetes and frequent PVC (defined as >10 PVC/hour) were more common in cardiac death/arrest patients. At multivariate analysis, a post-MI LVEF <50% at 8–10 weeks combined with TWA and HRT measured at 10-14 weeks improved outcome prediction. Of note, TWA and HRT were not predictive if assessed in the first 10–14 weeks after MI.

Post-MI systolic function recovery was associated with SCD also in the CARISMA study [27], where patients with no LVEF recovery (ΔLVEF ≤0%) showed a five-fold higher risk of cardiac mortality, compared to patients with any degree of LVEF recovery (9% vs. 2%, *p* = 0.031) [28].

Finally, this trend was also confirmed in the ISAR-Risk study [17] with no LVEF recovery being associated after a follow-up of 5 years with a two-fold higher risk of SCD (unadjusted HR 2.3; 95%CI: 0.9–6.3; *p* = 0.09) and an almost four-fold higher risk of all-cause mortality (unadjusted HR 3.6; 95% CI: 0.9–14.2; *p* = 0.07). Moreover, the SCD rate of patients with SAF and LVEF >30% was similar to that of patients with LVEF <30%. Compared to patients with LVEF <30%, patients with SAF and LVEF >30% were older (68 vs. 62 years; *p* = 0.0001) and more often female (32 vs. 12%; *p* = 0.0001). Risk stratification based on an LVEF cut-off of 30% identified only approximately 25% of SCD population, while the combination of LVEF <30% with LVEF >30% plus SAF identified approximately 50% of them.

### 3.3. Echocardiography

Echocardiography has been traditionally considered as the cornerstone to define the risk of cardiac mortality after MI [29] and the evaluation of LVEF is currently the most important parameter to guide ICD implantation according to international guidelines [6,7]. However, this approach has limitations: LVEF is a good predictor of the overall mortality but is a poor stratification tool for prediction of arrhythmic risk. Many patients with LVEF >35% may suffer from SCD [30], and many patients with LVEF <35% will never receive appropriate ICD shocks [31]. Nowadays, more sophisticated echocardiographic modalities may help to further stratify patients. For example, strain echocardiography evaluating mechanical dispersion and global strain showed to be a good predictor of ventricular arrhythmias after MI, particularly in patients with LVEF >35% [32]. This may improve the identification of patients with LVEF >35% who may benefit from ICD implantation, but that are not currently fulfilling classical guidelines criteria.

### 3.4. Cardiac Magnetic Resonance

There is growing appreciation of cardiac magnetic resonance (CMR) with late gadolinium enhancement (LGE) imaging to stratify prognosis and predict SCD risk across a variety of myocardial diseases and a wide spectrum of LVEF [33,34]. CMR with LGE can be used to identify and quantify areas of replacement fibrosis [35], a marker strongly related to infarct size, risk of maladaptive remodeling and a major substrate for reentrant arrhythmias [36]. In patients with known CAD, LGE was a better predictor of VT inducibility at electrophysiological study (EPS) than LVEF: an LGE threshold of 15% of LV mass showed a 89% sensitivity and 48% specificity for a positive EPS, while 10% of LV was the critical amount of fibrosis for VT inducibility [37]. Notwithstanding that EPS inducibility is a surrogate of SCD (highly dependent upon the protocol used and with a non-negligible false positive results rate), these findings furnish a pathophysiological link between fibrosis and arrhythmic re-entry [38]. Subsequent studies reinforced the role of LGE mass as an independent predictor of all-cause mortality [39]. Recently, the so called “grey zone fibrosis” mass defined on the basis of standard deviation from maximal signal intensity LGE was more strongly associated with SCD and VAs than LVEF [40].

The DETERMINE trial is evaluating the benefit of ICD on mortality in patients with CAD, LVEF >35% or LVEF 30–35% with no inducible VT and LGE ≥10% of LV mass [41].

There is scant evidence that the LGE pattern might also help in risk stratification as a midwall striae pattern of fibrosis was an independent predictor of sudden cardiac arrest or appropriate ICD therapy, and the result was consistent also in patients with LVEF >35% [42].

Risk stratification in the early phase of AMI remains challenging. Currently, the PROTECT-ICD trial (NCT 03588286) [43] is enrolling STEMI patients with LVEF ≤40% in the first 40 days after the event to assess the role of EPS to guide ICD implantation. This will be correlated with CMR study to assess its predictability on subsequent inducible VT at EPS and SCD or arrhythmia. Data on non-selected AMI population, including those with pLVEF, are scant. In a first, non-complicated STEMI population with a mean LVEF of 52% who underwent CMR within 1 week of presentation, low LVEF (≤36%) and CMR-derived infarct size (≥23.5 g/m^2^) best predicted adverse arrhythmic events [44]. Of note, most of these occurred in the population with severely reduced LVEF. Similarly, quantification of ischemic penumbra with CMR within 7 days of a first STEMI proved to be a useful tool to predict subsequent VT occurrence [45]. In this population with a mean LVEF of 42%, patients with larger ischemic penumbra areas, corrected for total LGE area size, had more often VTs (OR 1.05, 95% CI 1.01–1.10; *p* = 0.02). Interestingly, no interaction was measured between LVEF and percentage penumbra of total enhanced myocardium on VT development, and VT occurred similarly in patient with LVEF higher or lower than 40%. Overall, there is convincing evidence of the additive value of CMR studies for prognosis stratification in STEMI patients [46].

The role of CMR in NSTEMI setting is more debated. In a cohort of NSTEMI patients with a median LVEF of 51% (IQR 45–58) who underwent CMR 3 (IQR 2–4) days after presentation, no CMR-derived parameter of myocardial injury beyond LVEF was predictive of the composite of death from any cause, reinfarction and congestive heart failure at a follow-up of 4.4 years [47].

Beyond the acute AMI phase, a recent meta-analysis confirmed the predictive value of CMR quantification of the peri-infarction area in patients with ischemic cardiomyopathy as a predictor of subsequent appropriate ICD therapy, inducibility of VT at EPS and long-term mortality, but half of included studies enrolled only patients with LVEF ≤ 35% [21].

### 3.5. Nuclear Imaging

Nuclear imaging modalities, such as single photon emission computed tomography (SPECT) and positron emission tomography (PET), have been under evaluation for their ability to predict the risk ofVAs, independently from cardiac function.

SPECT was shown to be a good predictor of SCD in patients with known CAD and LVEF >35%, based on the degree of stress defect. In fact, patients who experienced SCD showed a higher summed stress score (SSS >8), defined as the sum of the relative stress perfusion defect in each myocardial segment [20]. Meta-iodo-benzyl-guanidine (MIBG) is a norepinephrine analog, and the signals of MIBG labeled with I-123 allow to visualize neuronal uptake evaluating abnormalities in the cardiac sympathetic nervous system. The evaluation by SPECT imaging of MIBG uptake in the border zone of MI-induced scars may help to predict VA inducibility at EPS [19]. Moreover, MIBG uptake can predict appropriate shocks in patients with ICD [48].

PET allows the combined evaluation of myocardial blood flow and metabolism; an increasing percentage of ischemic myocardium at 82-Rubidium PET appeared as a good predictor of cardiac death, non-fatal myocardial infarction and all-cause mortality [49].

Nuclear imaging techniques are promising as stratification tools for VAs, but no robust validations have been performed yet.

### 3.6. Electrophysiological Study

Current ESC guidelines [7] on the management of patients with ventricular arrhythmias and the prevention of SCD do not recommend any non-invasive risk stratification approaches because of low specificity and sensitivity, but EPS should be considered in myocardial infarction survivors with pLVEF and otherwise unexplained syncope (Class of recommendation IIa, C).

Gatzoulis et al. [50] evaluated the role of EPS as a guide to ICD implantation in the patients with LVEF >35%. Patients with mean LVEF of 43% (65% ischemic cardiomyopathy) were implanted in the presence of at least one of the following: NSVT detected by 24-h Holter ECG, history of syncope or VT inducibility at programmed ventricular stimulation. Using this approach, no difference in the cumulative incidence of appropriate ATP or shocks were observed between patients with LVEF > 35% and LVEF < 35%. Building on these premises, the PRESERVE EF study proposed a new two-step algorithm for risk stratification in post-MI patients with pLVEF. In the first step, patients with at least one non-invasive risk factor among PVC, NSVT, late potentials, prolonged QTc, increased TWA, abnormal HRT, reduced heart rate variability were referred for EPS (second step) and implanted (in most cases) in case of inducible malignant arrhythmia (monomorphic VT, ventricular flutter or polymorphic VT). In this study, no SCD occurred in patients without risk factors and in those with a negative EPS study (negative predictive value 100%), while ICD intervention occurred in 22% of the inducible population (specificity 93.8%), with an annual incidence rate of 8.2% [51]. Patients with ≥2 risk factors were more likely to be inducible compared with those with a single factor (OR ≥ 2 factors/factor = 1 = 2.5, 95% credibility interval 1.2–5.5, *p* = 0.02). Late potentials and NSVT were more frequently present in inducible, than in non-inducible patients (51.2 vs. 31.5%, *p* = 0.036; 46.3 vs. 23.1%, *p* = 0.009, respectively). No events occurred in patients with an LVEF > 50%. Ongoing trials on SCD risk stratification in patients with pLVEF are summarized in Table 2.

## 4. Risk Stratification of SCD in Patients with HFpEF

In the setting of patients with HFpEF the CHARM-preserved trial [52], evaluating the angiotensin receptor blocker candesartan, reported a rate of SCD of 1.5/100 patient years accounting for about 8.5% of all-cause mortality (and 35% of cardiovascular deaths). In the I-PRESERVE [10], the SCD rate was 1.38 deaths/100 patients per year (26% of all-cause deaths), and 18 out of 169 HFpEF patients experienced SCD in the JCARE-CARD [53] (11% of all-cause deaths). The observed discrepancies in SCD contribution to death events can be to some extent attributed to differences in the populations enrolled: in the CHARM-Preserved trial, 35% of patients had LVEF ≥ 50%, as compared to 12% in the I-PRESERVE study; furthermore, the latter enrolled fewer patients with ischemic heart disease. In the most recent TOPCAT trial [54], including patients with LVEF ≥ 45% and hospitalization for heart failure in the preceding 12 months, SCD had an incidence of 1.4 events/100 patient years (accounting for 19% all-cause deaths). Despite different prevalence of SCD in these different studies, a reproducible SCD rate of approximately 1.4 events/100 patient years emerges in a population of preserved LVEF.

**Figure 2:**
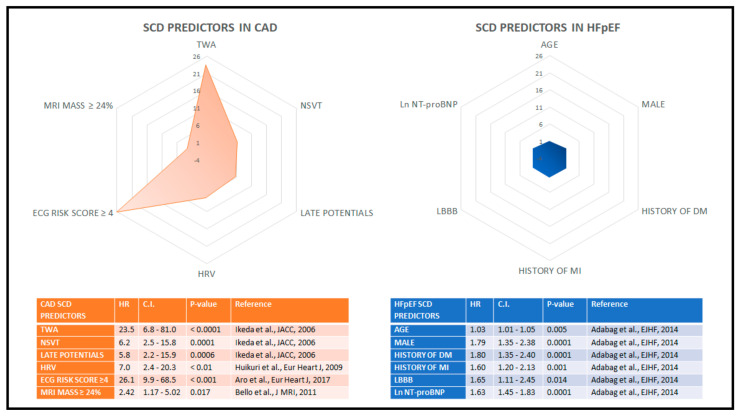
Predictors of sudden cardiac death. The radar graphs show the relative potency of the SCD predictors in terms of hazard ratios, in the context of CAD with preserved ejection fraction (left panel) and in HFpEF (right panel). Predictors of sudden cardiac death display a higher overall predictive power in CAD compared to HFpEF patients, as highlighted by the discrepancy between the colored areas in the polar grid, which can be interpreted as the amount of risk forecastable by available variables. CAD: coronary artery disease; HFpEF: heart failure with preserved ejection fraction.

The current strategy for risk stratification in patients with HFpEF is still not well defined, when compared to that of CAD patients (Figure 2). In the I-PRESERVE trial, patients with age ≥75 years, NT-proBNP ≥339 pg/mL, presence of diabetes mellitus, previous hospitalization for HF and NYHA class IV showed a significant increase in event rate, including SCD. Women had significantly lower rates of events, compared to men. Furthermore, the lowest LVEF quartile (≤52%, IQR 5%) had significantly higher rates of SCD, compared with the other three quartiles [10]. Diabetes mellitus seems to play an important role in the SCD risk of patients with HFpEF. Using the Duke University databank for cardiovascular disease, Al-Khatib et al. [55] found that five clinical variables were predictors of sudden cardiac death (SCD) in patients with isolated diastolic HF: diabetes mellitus, mitral regurgitation, severity of symptoms as assessed by NYHA class, history of MI and severity of CAD(number of diseased coronary arteries). In a sub-analysis of the I-PRESERVE trial, the following variables were associated to a ≥10% risk of SCD over 5 years at multivariable analysis: age, gender (male), history of diabetes mellitus, history of myocardial infarction, left bundle branch block (LBBB) and log_e_ NT-proBNP [56]; however, to date there is no trial demonstrating a benefit of ICD in the HFpEF population.

Similar results were obtained by a sub-analysis of the TOPCAT trial [54], after accounting for competing risks of non-SCD: only male sex and insulin-treated diabetes mellitus identified patients at higher risk for SCD, although with modest discrimination (C-statistic = 0.65); age, LVEF, CAD, LBBB and baseline therapies were not independently associated with SCD. Recently, an impaired LV global longitudinal strain was also found to be an independent predictor of SCD [57]. Finally, in the JCARE-CARD registry, the only predictor of SCD in HFpEF was lower eGFR (HR 1.026; CI 1.001–1.051 *p* = 0.040).

## 5. Risk Stratification of SCD in Patients with Acute Myocarditis

Myocarditis is an inflammatory disease of the myocardium, generally caused by a viral infection but possibly encompassing other potential immunological triggers [58]. The annual rate of acute myocarditis is estimated to be 22 cases/100,000, with this figure possibly being underestimated due to physicians underreporting mild cases with vague symptoms [59]. Autopsy series have consistently shown acute myocarditis as the cause of SCD in approximately 3–12% of subjects with less than 40 years of age [60,61,62]. On the other hand, the incidence of SCD in the myocarditis population is currently unknown, due to the lack of dedicated studies. In a recent study comprising 443 subjects with acute myocarditis, the 35-month cumulative incidence of cardiac death and sustained ventricular tachycardia (VT) was 0.5 and 0.9%, respectively [63]. Interestingly, all events occurred in subjects presenting acutely with complicated myocarditis, defined as myocarditis associated with at least one among: LVEF < 50%, ventricular arrhythmias or low cardiac output syndrome; however, the study did not report outcomes stratified by LVEF [63]. In a larger cohort of 670 subjects with acute or sub-acute myocarditis (i.e., symptoms persisting for more than 2 weeks), death occurred in 29 (4%) subjects and sustained VT in 22 (3%) subjects at a median follow-up of 4.7 years. The annualized event rate for major adverse cardiovascular events (including death from any cause, sustainedVT, heart transplantation or recurrent myocarditis) among the 470 patients with LVEF > 40% was 1.1% if no late gadolinium enhancement (LGE) could be identified on cardiac magnetic resonance (CMR) and 2.6% in the presence of LGE [64]. Indeed, the presence of LGE, aside being included among the diagnostic criteria for myocarditis, appears to be associated with adverse outcomes specifically in myocarditis patients with pLVEF. In a cohort of 374 myocarditis patients with LVEF > 50% on CMR, SCD was observed in four subjects, with two further resuscitated cardiac arrest and two cases of appropriate ICD shock, at a median follow-up of 1572 days. All subjects with the reported events had anteroseptal LGE accumulation [65]. This was found to be consistent with post-mortem studies of myocarditis patient with SCD, in whom heavy inflammatory cells burden within the interventricular septum could be readily identified [66]. Aside from imaging prognosticators, several ECG parameters known to predict malignant arrhythmias and SCD in diverse cardiomyopathies have been evaluated in the setting of myocarditis. In a cohort of 186 subjects with acute myocarditis the presence of at least one ectopic ventricular beat on the admission ECG, wide QRS complex and long QT segment were associated to cardiac death independently of LVEF at a median follow-up of 55 months [67]. The relevance of QT interval in identifying subjects at increased risk for ventricular arrhythmias has been recently confirmed in a small report on 56 subjects with acute myocarditis and preserved LVEF [68]. The investigators also reported an increased T wave peak-to-end interval in subjects with sustained ventricular arrhythmias at follow-up. The role of ICD insertion in myocarditis patient is not well defined and is currently recommended mainly as secondary prevention in the subacute phase of the disease [30]. In subjects with documented heart rhythm disturbances, a wearable cardioverter defibrillator is an attractive option until either the risk resolves or requirements for ICD implantation are met [69]. Specific etiologies for myocarditis forms deserve a special mention when it comes to SCD risk. Cardiac sarcoidosis is an inflammatory cardiomyopathy associated with high rate of atrioventricular block and sustainedVT; as a result, SCD in this cohort may be the consequence of either tachy- or bradyarrhythmias [70]. Very few data on SCD risk stratification exist in these setting, and currently, the incidence of SCD in cardiac sarcoidosis with preserved LVEF is not known. LGE detection with CMR and subsequent evaluation with EPS is currently advised to inform the indication for ICD insertion [71]. Giant cell myocarditis is a highly malignant form of myocarditis associated with ventricular arrhythmias in approximately one third of cases and an overall dismal prognosis [72]. Lyme disease-associated myocarditis frequently causes advanced atrioventricular block without substantial cardiac dysfunction and can infrequently cause SCD [73]. Cardiac involvement in Chagas disease manifests as conduction disturbances, high degree atrioventricular block and sustained or non-sustained ventricular arrhythmias before causing dilated cardiomyopathy or apical aneurysm [74]. SCD is the main cause of death in Chagas patients, accounting for approximately 40–70% of deaths [75]. It has been described as secondary both to bradyarrhythmia or tachyarrhythmia and can occur early in the course of the disease, when heart function is still preserved. No reliable SCD risk prediction model currently exist, and thorough clinical evaluation and ambulatory ECG monitoring are advocated to identify high risk individuals [75,76].

## 6. Risk Stratification of SCD in Patients with Atrial Fibrillation

AF is common in the population with LVEF >45%, especially in the HFpEF cohort, where it may be as high as 30–40% [12,77]. Atrial fibrillation may increase the risk of SCD [78], and SCD is the leading death cause in the AF population [79]. In a large population-based cohort of patient with a long-term (>10 years) follow-up and low HF prevalence (<5%), incident AF more than doubled the risk of SCD with an HR of 2.47 (1.95–3.13; *p* < 0.001), independently of other variables [80]. Similar results were confirmed in a cohort of hypertensive patients with a very low prevalence of HF, where AF increased the risk of SCD by three-fold and in a case-control study of patients with out of hospital cardiac arrest [81,82].

While these data reinforce the link between AF and SCD, they do not provide mechanistic insight on a potential causative effect of AF on VT/VF. Recently, temporal association between episodes of atrial high-rate episodes (AHRE) and VT/VF has been demonstrated in a population of severely reduced LVEF patients and already implanted ICD/CRT-D devices. An AHRE episode increased the risk of subsequent VT/VF (with OR ranging from 1.84 to 3.06) in a 30-day time interval, and VT/VF occurred simultaneously with AHRE in 85% cases; patients who developed AHRE <48 h before VT/VF also showed higher ventricular arrhythmias recurrence rates and mortality [83]. In general, AF may be the trigger for malignant ventricular tachyarrhythmias due to: reduction in ventricular refractoriness during rapid heart rate and beat-to-beat variation in cycle length, worsening of cardiac output, myocardial ischemia, activation of sympathetic tone and probably a combination of these factors [78,84].

Risk factors for SCD in the AF population can be deduced by some of the previously cited studies, but the amount of relevant data is scant. In the hypertensive population, presence of the Sokolow–Lyon criteria for left ventricle hypertrophy raised the risk of SCD in the cohort with incident AF [82]. In this same population, black people tended to show a greater risk of SCD: this was confirmed in the ARIC study, where the risk of SCD associated with AF was higher in blacks (HR 5.77 vs. 2.49; *p* for ethnicity interaction = 0.02) [80].

Taken together, these data suggest a pathophysiologic connection between AF and SCD, but death risk stratification in the general AF population remains limited along with a limited comprehension of the underlying causative mechanism.

## 7. Emerging Risk Factors: Genetics, Biomarkers and Obstructive Sleep Apnea

Currently, there is no consensus on the use of genetic testing for unexplained SCD. This is mainly related to the costs and the issues in the interpretation of variants of unknown significance (VUS) [85]. The costs of genetic testing are undergoing a significant reduction, due to the availability of next-generation sequencing and panels with large numbers of genes.

The Cardiac Arrest Survivors with Preserved Ejection Fraction Registry (CASPER) is a large registry of SCD survivors without signs of SHD [86]. From 2006 to 2015, 174 out of 375 SCD survivors underwent genetic testing. A pathogenic variant was identified in 17% individuals, while at least one VUS was identified in 18% patients.

Compared to genetic analysis, biomarkers might be cost effective, readily available and with lower interpretation issues [87]. In the Physicians’ Health Study, C-reactive protein (CRP) levels were an independent risk factor for SCD in males [88]. Interleukin-6 was associated with SCD in the PRIME study [89]. Other inflammatory markers emerging as SCD predictors are von Willebrand factor, factor VIIIc and fibrinogen [90]. Hemodynamic markers such as NT-proBNP were shown to predict risk of SCD both in CAD and heart failure [91]. Other new biomarkers of SCD are magnesium [92], which is a membrane stability regulator whose levels are inversely associated with SCD, and cystatin C, a marker of renal failure, inflammation and atherosclerosis [93]. The main restraint to the use of biomarkers in clinical practice is their moderate increase in risk prediction; validation in large population samples is still to come [87].

Disorders of sleep, such as obstructive sleep apnea (OSA), insomnia, abnormal sleep duration and poor sleep quality have been associated with cardiovascular disease (CVD) morbidity and mortality [94,95,96]. OSA, despite being underdiagnosed, is by far the most common form of sleep apnea, affecting 9–38% of the global adult population; its prevalence increases with body weight, age and male gender [97].

Systemic hypoxia due to OSA contributes to the subendocardial ischemia; the latter sets the stage for the structural and electrical remodeling known to predispose to SCD [98]. Additionally, OSA-induced intermittent hypoxia contributes to increase the sympathetic tone through chemoreceptor and baroreceptor triggering, along with catecholamine release [99]. Repeat apneas and awakenings over time can alter normal hemodynamics and cause inflammatory disturbances; the resulting cardiac remodeling can be a substrate for VA independent from the mechanism acutely causing VA during OSA.

There are multiple ECG markers of increased risk of SCD associated with OSA [98,99,100,101]. These include PVCs, increased HRT, QT interval prolongation, AF and TWA [102]. Atrioventricular block has also been shown to be a frequent rhythm disturbance in OSA [103]. Individuals with severe OSA have a higher risk of nocturnal non-sustained VT and complex ventricular ectopy [104]. Patients with these ECG abnormalities have a two-fold increase in SCD during sleep [105]. A 2018 meta-analysis found that CPAP treatment might prevent subsequent cardiovascular events; CPAP was associated with a significantly lower risk of major adverse cardiovascular events in six of seven observational studies (RR, 0.61; 95% CI: 0.39–0.94, *p* = 0.02) [106].

## 8. Conclusions

Risk stratification in patients with pLVEF, although relevant for the prevention of SCD, is still hampered by several difficulties. A light in the shade is provided by the PRESERVE EF study [46], which proposed a fascinating two-step algorithm for patients with ICM and pLVEF. However, before applying the model in current clinical practice, we have to weigh the possible advantages against the increased numbers of invasive procedure. Based on 2005–2014 ARIC study [23], 720,000 acute MI per year are expected in the US, with 77% of them in presence of LVEF > 35%, accounting for 554,400 MI patients per year with pLVEF. Clinical application of the PRESERVE EF algorithm would lead to EPS in 194,040 patients per year, with 52,390 of them inducible for malignant arrhythmia. Given that 22% of implanted patients in PRESERVE EF had a major arrhythmic event, 11,525 patients per year in the US would receive an appropriate treatment from the ICD; if applied in clinical practice, this would lead to a further 35% increase to the 150,000 ICDs annually implanted in the United States [107]. Additionally, among considered risk factors, only NSVT and late potentials were more frequent in inducible patients; no events occurred in patients with an LVEF >50%. Further analysis should evaluate if limitation of EPS only to patients with LVEF ≤50% and the use of weighted risk scores instead of the mere presence of prespecified risk factors could provide effective stratification without increasing the number of EPS and ICD implants.

Effective risk stratification in patients with HFpEF is hampered by the substrate heterogeneity and future studies including big data from well-characterized population might improve our understanding.

## Figures and Tables

**Table 1:** Studies evaluating the relationship between left ventricular ejection fraction and sudden cardiac death.

Study	Year	CAD	Number of Patients	Baseline Characteristics			FU (y)	Clinical Variables Associated with a Higher Proportional Risk of SCD *
				Age (y)	Women (%)	LVEF (%)		
**Hsia et al. [16]**	2008	YES	8290	63.9	18	58	4.8	- Age - Current angina pectoris - LVEF 40–50% - Duretic use - Digital use
**PRE-DETERMINE [8]**	2018	YES	5761	64	34	52	3.9	- Reduced LVEF - Age - NYHA class
**ISAR-R [17]**	2009	YES	142	68	37	46	5	- Severe autonomic failure
**Ikeda et al. [18]**	2006	YES	1041	64	31	55	2.7	- Positive microvolt TWA
**I-PRESERVE [10]**	2010	NO	4128	72	60	59	5	- Age ≥ 75 years - NT-proBNP ≥339 pg/mL - Diabetes mellitus - Previous hospitalization for HF - NYHA class IV - Lower LVEF
**Al-Khatib et al. [19]**	2007	NO	1941	65	55	63	3.9	- Diabetes mellitus - MR - Severity of symptoms - History of MI - Severity of CAD
**TOPCAT [20]**	2018	NO	1767	68.6	52	-	3	- Male - Insulin-treated diabetes mellitus
**JCARE-CARD [21]**	2012	NO	169	77.5	45	57.5	2.1	- Lower eGFR

Age, LVEF and FU are reported as means. * *p* < 0.05. FU (y) = years of follow-up; LVEF = left ventricle ejection fraction; TWA = T wave alternans; HF = heart failure; CAD = coronary artery disease; eGFR = estimated glomerular filtration rate; MR = mitral regurgitation; MI = myocardial infarction.

**Table 2:** Ongoing trials on sudden cardiac death in patients with preserved ejection fraction.

Trial	Complete Title	Principal Investigator	Study Design	Estimated Enrollment (Patients)	Aim of the Study	Estimated Study Completion Date
**PROFID-Preserved**	Personalised Risk Score for Implantation of Defibrillators in Patients with Preserved LVEF > 35% and a High Risk for Sudden Cardiac Death	Gerhard Hindricks, MD	Non-commercial, investigator-driven, prospective, parallel-group, randomized, open-label, blinded outcome assessment, multi-center, superiority trial	1440	The objective of the study is to demonstrate that in post-MI patients with preserved LVEF > 35% but high risk for SCD according to a personalized risk score, the implantation of an index group (ICD) is superior to optimal medical therapy (control group) with respect to all-cause mortality.	31 December 2024
**ReCONSIDER Study**	Arrhythmic Risk Stratification in Nonischemic Dilated Cardiomyopathy	Konstantinos A Gatzoulis, MD	Prospective observational multicenter	675	This trial aim to integrate several approaches to arrhythmic risk stratification in nonischemic dilated cardiomyopathy in patients with preserved LVEF > 35% in a tiered, multifactorial, approach, in which noninvasive risk factors are combined with electrophysiologic studies.	1 May 2025
**SMART-MI**	Implantable Cardiac Monitors in High-Risk Post-Infarction Patients with Cardiac Autonomic Dysfunction	Axel Bauer, MD; Stefan Kaeaeb, MD	Randomized, interventional trial with parallel assessment	400	There is a large body of evidence that presence of cardiac autonomic dysfunction is associated with an increased susceptibility to malignant brady- and tachyarrhythmias eventually culminating in SCD in post-MI patients with LVEF >35%. SMART-MI will assess the occurrence and prognostic implications of serious arrhythmic events in this newly identified high-risk group by remote monitoring with ICM.	July 2021

## Data Availability

Data sharing not applicable.
